# The complete mitochondrial genome of the hybrid of *Haliotis discus hannai* (♀) × *Haliotis Iris* (♂)

**DOI:** 10.1080/23802359.2019.1669089

**Published:** 2019-09-25

**Authors:** Zhansheng Guo, Yi Ding, Li Nian, Zhaoyang Jiang, Zhenlin Liang, Xuguang Hou

**Affiliations:** Marine College, Shandong University at Weihai, Weihai, China

**Keywords:** Hybrid, *Haliotis discus hannai*, *Haliotis iris*, mitogenome

## Abstract

In this study, the complete mitochondrial genome of the hybrid of *Haliotis discus hannai* (♀) × *Haliotis iris* (♂) was sequenced and analyzed for the first time. The mitogenome had a length of 16,719 bp and contained 13 protein-coding genes (PCGs), 2 rRNA genes, 22 tRNA genes, and 1 control region (CR). The sequence identity of the complete mtDNA sequences between the hybrid F1 and *H. discus hannai* was 99.40%, which revealed that the mitogenome of the hybrid subjected to the maternal inheritance rule. The phylogenetic analysis also presented that the hybrid F1 was relatively more close to *H. discus hannai*.

*Haliotis discus hannai*, endemic to northeast Asia, is an economically important aquaculture mollusc (Guo, Ding, et al. 2019). Owing to the lack of effective and scientific management, the cultured population gradually exhibits disease susceptibility, growth depression, and early sexual maturity. To improve abalone production, *Haliotis iris* have been introduced into China. The hybrid F1 generation was obtained by interspecific breeding, *H. discus hannai (*♀) × *H. iris (*♂) (Guo et al. 2013), and the complete mitogenome of the hybrid F1 was sequenced for the first time.

The hybrid F1 was collected from Chinese National Engineering Center of Marine Shellfish (37°10′20″N, 122°33′4″E), China. The voucher specimen was deposited at the Key Laboratory of Marine Breeding, Shandong University, Weihai, China (SDU15082401). Total genomic DNA was extracted using the TIANamp Marine Animals DNA Kit (TianGen Biotech Co., Ltd, Beijing, China) according to the manufacturer’s instructions. Twenty-four pairs of primers were designed to amplify the entire mitogenome. The mitogenome sequence was assembled and annotated according to a previously described procedure (Su and Liang 2018; Ahmad et al. 2019).

The complete mitogenome of the hybrid abalone has been deposited in the GenBank under accession number KU310897. The mitogenome had a length of 16,719 bp and contained 37 genes (13 protein-coding genes, 2 rRNA genes, 22 tRNA genes) and 1 control region (CR). The overall nucleotide composition was A (35.4%), T (24.9%), C(26.1%), G (13.6%), and the values of AT skew and GC skew were 0.173 and −0.313, respectively. The gene arrangement and transcriptional direction are similar to other *Haliotis* species (Guo, Ding, et al. 2019; Guo, Jiang, et al. 2019). Seven PCGs including *cox1-3, nad2, nad3, atp6*, and *atp8* were encoded on heavy strand and the rest six PCGs were encoded on light strand. Twenty-two identified tRNA genes had a total length of 1505 bp, 16S rRNA, and 12S rRNA were located between tRNA^Leu2^ and tRNA^Val^ and between tRNA^Val^ and tRNA^Met^ and had lengths of 1453 and 1009 bp, respectively. The sequence identity of the complete mtDNA sequences between the hybrid F1 and *H. discus hannai* and between the hybrid F1 and *H. iris* were 99.40 and 83.98%, respectively. The organizations of hybrid F1 were quite similar to their female parent and subjected to the maternal inheritance rule (Zhang et al. 2016).

Fifteen *Haliotis* mitogenomes available in GenBank were subjected to phylogenetic analyses, using *Lunella granulata* (NC 031857) as the outgroup. The nucleotide sequences of 13 PCGs were extracted using PhyloSuite (Zhang et al. 2018) and aligned using MAFFT (Katoh and Standley 2013). These alignments were then concatenated to yield the data set using PhyloSuite (Zhang et al. 2018). Phylogenetic analyses were conducted using the maximum likelihood (ML) using MEGA X (Kumar et al. 2018) with 1000 replicates. The phylogenetic tree included two major clusters (Figure 1). During tree formation, *H. discus hannai* and the hybrid F1 initially clustered together and then clustered with *H. iris*.

**Figure 1.**
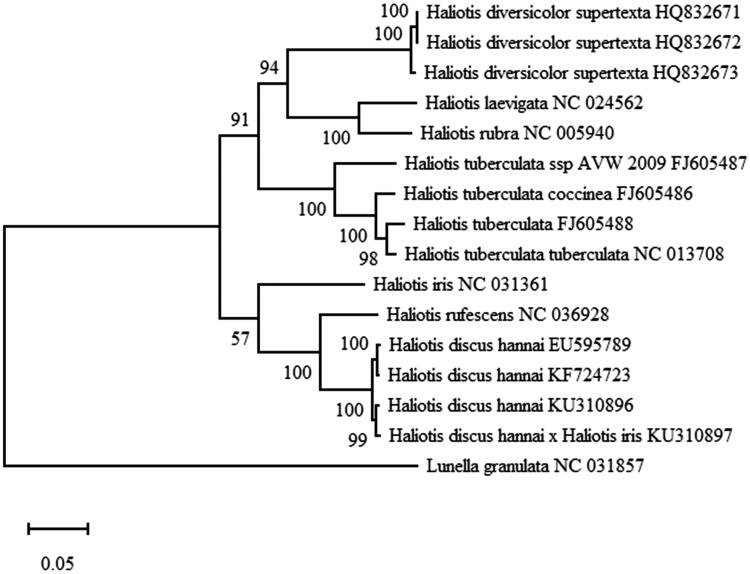
The phylogenetic analysis of related *Haliotis* species based on the 13 PCGs using maximum likelihood method (ML) with 1000 replicates.
